# Idelalisib and bendamustine combination is synergistic and increases DNA damage response in chronic lymphocytic leukemia cells

**DOI:** 10.18632/oncotarget.15180

**Published:** 2017-02-07

**Authors:** Prexy Modi, Kumudha Balakrishnan, Qingshan Yang, William G. Wierda, Michael J. Keating, Varsha Gandhi

**Affiliations:** ^1^ Department of Experimental Therapeutics, The University of Texas MD Anderson Cancer Center, Houston, TX, USA; ^2^ Department of Leukemia, The University of Texas MD Anderson Cancer Center, Houston, TX, USA

**Keywords:** CLL, bendamustine, idelalisib, DNA damage, B cell receptor

## Abstract

Idelalisib is a targeted agent that potently inhibits PI3Kδ which is exclusively expressed in hematological cells. Bendamustine is a well-tolerated cytotoxic alkylating agent which has been extensively used for treatment of chronic lymphocytic leukemia (CLL). Both these agents are FDA-approved for CLL. To increase the potency of idelalisib and bendamustine, we tested their combination in primary CLL lymphocytes. While each compound alone produced a moderate response, combination at several concentrations resulted in synergistic cytotoxicity. Idelalisib enhanced the bendamustine-mediated DNA damage/repair response, indicated by the phosphorylation of ATM, Chk2, and p53. Each drug alone activated γH2AX but combination treatment further increased the expression of this DNA damage marker. Compared with the control, idelalisib treatment decreased global RNA synthesis, resulting in a decline of early-response and short-lived *MCL1* transcripts. In concert, there was a decline in total Mcl-1 protein in CLL lymphocytes. Isogenic mouse embryonic fibroblasts lacking *MCL1* had higher sensitivity to bendamustine alone or in combination compared to *MCL1* proficient cells. Collectively, these data indicate that bendamustine and idelalisib combination therapy should be investigated for treating patients with CLL.

## INTRODUCTION

Chronic lymphocytic leukemia (CLL) is a malignancy that is driven by active B-cell receptor (BCR) pathway. The primary BCR down-stream network includes two pivotal enzymes, Bruton's tyrosine kinase and phosphatidyl inositol three kinase (PI3K). Both BTK and PI3K isoform delta are selectively expressed in hematopoietic cells specifically normal and malignant B-cells such as CLL lymphocytes. This exclusive expression provided an impetus to design potent and selective inhibitors of these two proteins. Ibrutinib, a BTK poison and idelalisib a PI3K delta antagonist were tested in CLL and demonstrated on-target effect during preclinical experimentations and efficacy during clinical trials. Both drugs were FDA approved for patients with CLL.

As described above, PI3K delta isoform is pivotal in the BCR axis [1] and is selectively blocked by idelalisib [2]. Idelalisib promotes apoptosis in CLL by disrupting molecular pathways related to BCR signaling. Furthermore, idelalisib blocks signals from the microenvironment, tumor cell and microenvironment interactions, and mitigates pseudo-emperipolesis [3–6]. Clinically, as a single agent during phase I investigations, the drug has been well-tolerated and has prolonged survival of patients with CLL [2]. In combination with rituximab, during phase II clinical trial, the efficacy trend was maintained and this resulted in approval of the drug for relapsed/refractory CLL disease [1, 2, 7, 8].

In the front-line setting, the idelalisib and rituximab combination was tested for older patients with CLL [9]. Similarly for another study, treatment-naïve CLL patients were treated with idelalisib for two months as monotherapy followed by combination with ofatumumab [10]. In both these studies, responses were observed however with a toxicity profile which was much worse than what was observed in the relapsed/refractory disease. The toxicity was identified as immune-mediated and hence was more pronounced in front-line treatment [10, 11].

From phase I, phase II in previously treated CLL, and phase II in newly-diagnosed CLL, a few points emerged. First, while the drug was well-tolerated during earlier studies, in treatment naïve patients, there was unacceptable toxicity profile. Second, responses were mostly partial and complete remissions were limited. Third, in both cohort of patients (previously untreated or treated), similar to other BCR pathway antagonists, there was egression of CLL cells from lymph node which remained in peripheral blood. Finally, there is a need to combine idelalisib with CLL-specific agents that may result in deeper responses without much untoward toxicity.

Chlorambucil, cyclophosphamide, and bendamustine are three alkylating agents that have been used for decades for treatment of CLL. Of these three, regarding potency in the clinic, the cyclophosphamide is the strongest, followed by bendamustine, and chlorambucil. However, cyclophosphamide results in high untoward toxicity, a feature not favorable to combine with idelalisib. Bendamustine not only possesses alkylating agent properties but is also well-tolerated and FDA approved for patients with CLL along with established treatment recommendations. [12, 13].

Previously, we have demonstrated utility of bendamustine in preclinical setting for primary CLL cells and shown synergistic or additive interactions with fludarabine [14]. Importantly, both p53 positive and p53 mutated CLL responded similarly to bendamustine [15, 16]. The mechanism of CLL lymphocyte death elicited by bendamustine was due to DNA damage and repair response. Because PI3K inhibitors, including idelalisib also result in DNA damage, we postulated that combination of idelalisib to bendamustine may result in additive or synergistic cytotoxicity due to enhanced DNA damage. When used alone, bendamustine was more active in suspension cultures of CLL but stromal microenvironment protected CLL cells from bendamustine-mediated cytotoxicity [14]. One of the primary pathways of microenvironment-induced CLL cell survival is BCR axis that includes the PI3K/Akt cassette. Because idelalisib mitigates the BCR nexus, we hypothesized that microenvironment-induced resistance to bendamustine cell death of CLL lymphocyte may be abrogated with idelalisib addition.

In the current project, we tested that idelalisib-mediated suppression of BCR signaling would sensitize CLL cells to bendamustine, and this mechanism-based combination may lead to a synergistic interaction. We evaluated the cytotoxicity induced by idelalisib or bendamustine alone or in combination in primary CLL cells and the impact of this combination on DNA damage response and Bcl-2 family survival protein levels which are regulated by PI3K/Akt pathway. Our data demonstrate that this combination is synergistic, and we suggest a mechanism by which idelalisib increased the cytotoxicity of bendamustine.

## RESULTS

### Idelalisib and bendamustine combination is synergistic

**Figure 1:**
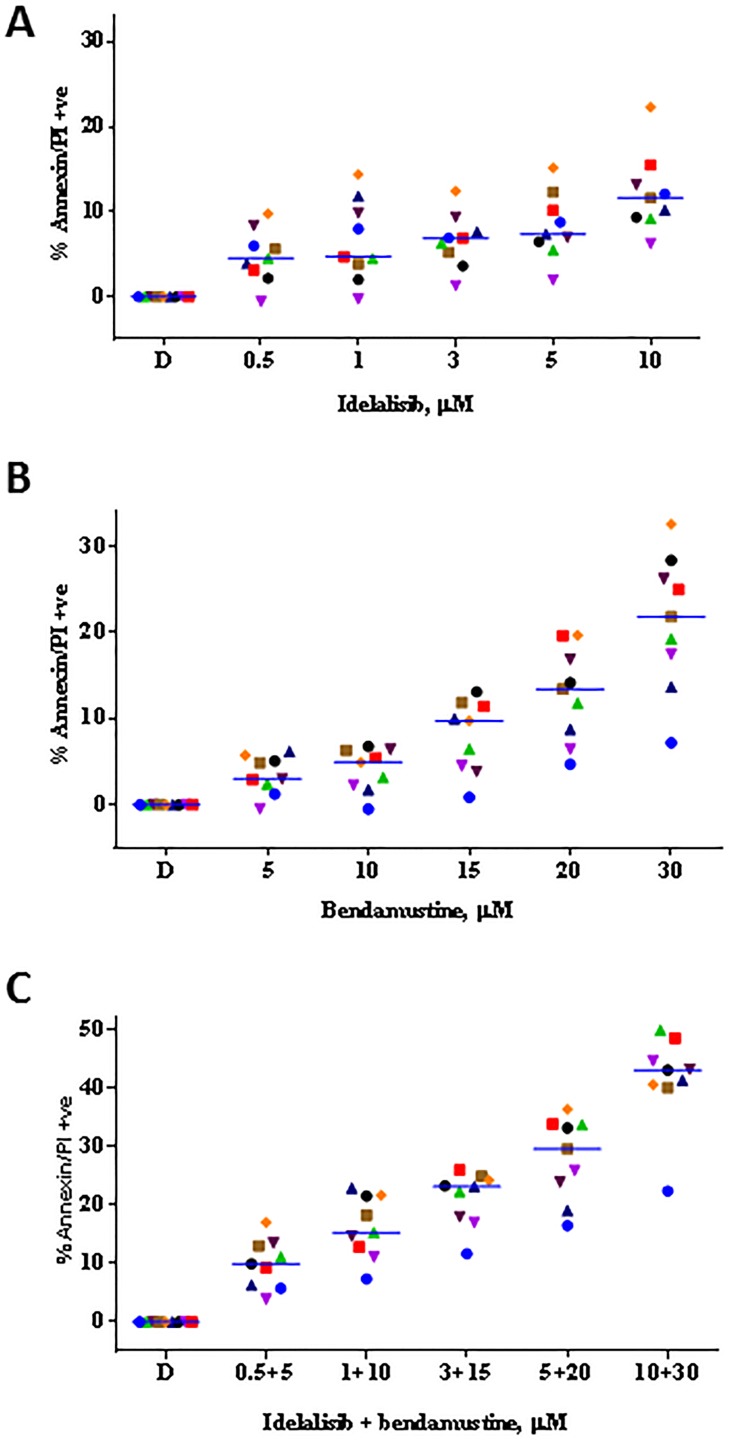

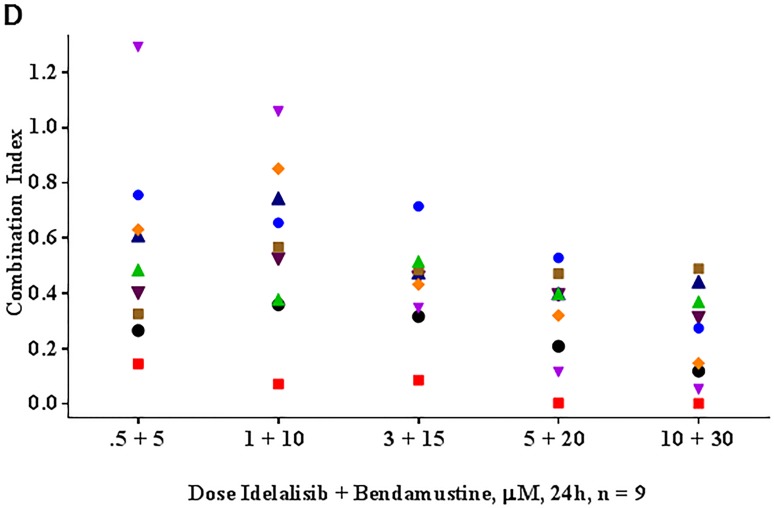
Idelalisib and bendamustine combination results in synergistic cytotoxicity **A**. Dose-dependent cytotoxicity of idelalisib alone or **B**. bendamustine alone or **C**. both in combination. Freshly isolated chronic lymphocytic leukemia (CLL) cells from 9 patients were incubated with the indicated concentrations of single agent idelalisib **A**., single agent bendamustine **B**., or both agents **C**. simultaneously for 24 hours. Cells were harvested and then stained with Annexin V/PI. The level of apoptosis for each treated sample was detected using flow cytometry, and the apoptotic cells were indicated by the cell population in the lower right, upper right, and upper left quadrants. The values were represented as percent control to untreated time matched control (DMSO; D) samples. Each colored symbol represents a CLL patient sample (CLL516, CLL944, CLL267, CLL109, CLL973, CLL781, CLL247, CLL661, and CLL112). Horizontal bar for each concentration represents median value. **D**. Evaluation of the combination index (CI) for the idelalisib and bendamustine combination. The apoptotic population from the combination treatment **C**. was used for fractional analysis. Calcusyn software was used for an output of the CI calculated by the fraction affected and the non-constant ratios of the drugs. The calculated combination index (CI) < 1, = 1, and > 1 indicate synergistic, additive, and antagonistic interactions, respectively. Abbreviation: h, hours.

Treatment of primary CLL cells with idelalisib alone (Figure 1A), bendamustine alone (Figure 1B), or the combination for 24 hours (Figure 1C; *n* = 9) revealed that single agent idelalisib induced moderate but statistically significant cytotoxicity (i.e., apoptosis) in a dose-dependent manner, ranging from 4% to 16% (*p* = 0.002 to < 0.0001 for each concentration). Whereas single agent bendamustine resulted in 6%-33% (*p* = 0.002 to < 0.0001 for each concentration) cell death. Combination of idelalisib and bendamustine at clinically relevant concentrations resulted in 13% to 49% of apoptosis (*p* = < 0.0001 for each concentration). At each drug concentration, compared to single agent apoptotic response, there was an increase in the level of cell death when both drugs were combined together.

This dose-response profile led us to investigate whether idelalisib and bendamustine combination could be synergistic combination. To test this, we plotted the apoptotic values obtained from Figure 1C and analyzed by the median-effect method using CalcuSyn software. The calculated combination index (CI) was < 0.8 for all the samples (except for 1 sample treated with low concentrations of both drugs) indicating synergy (Figure 1D).

### DNA damage response was enhanced with combination of idelalisib and bendamustine

**Figure 2:**
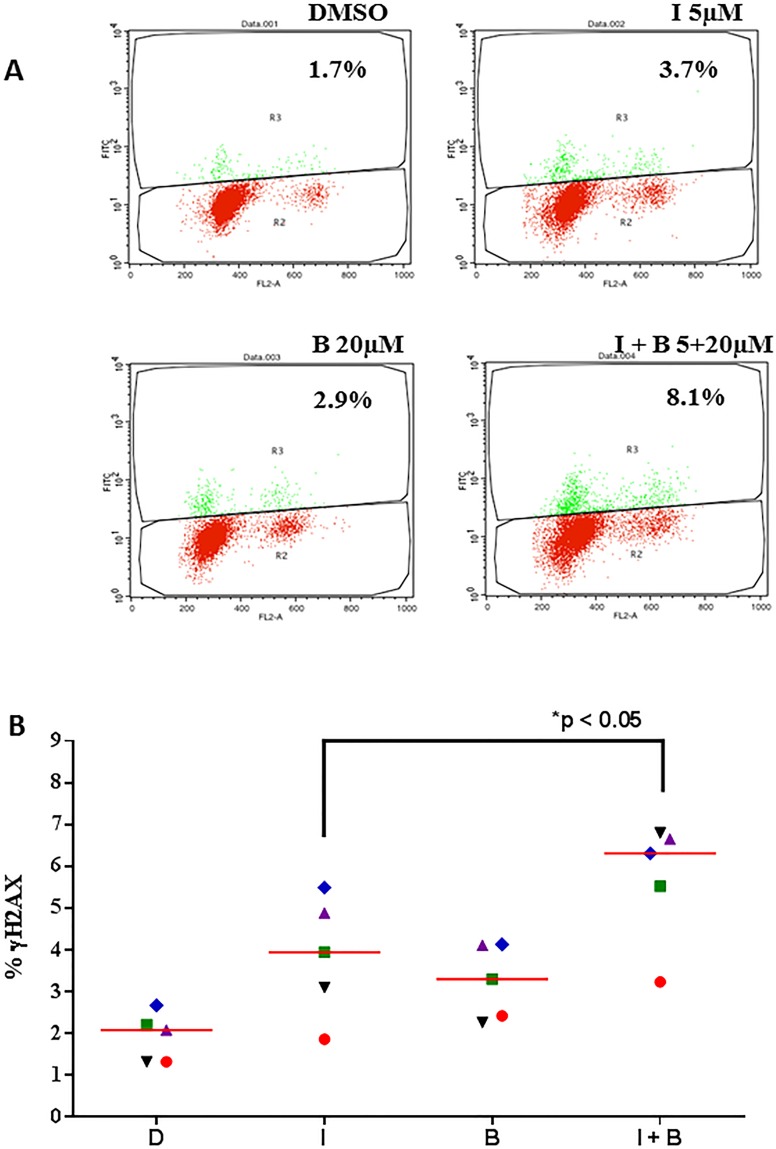

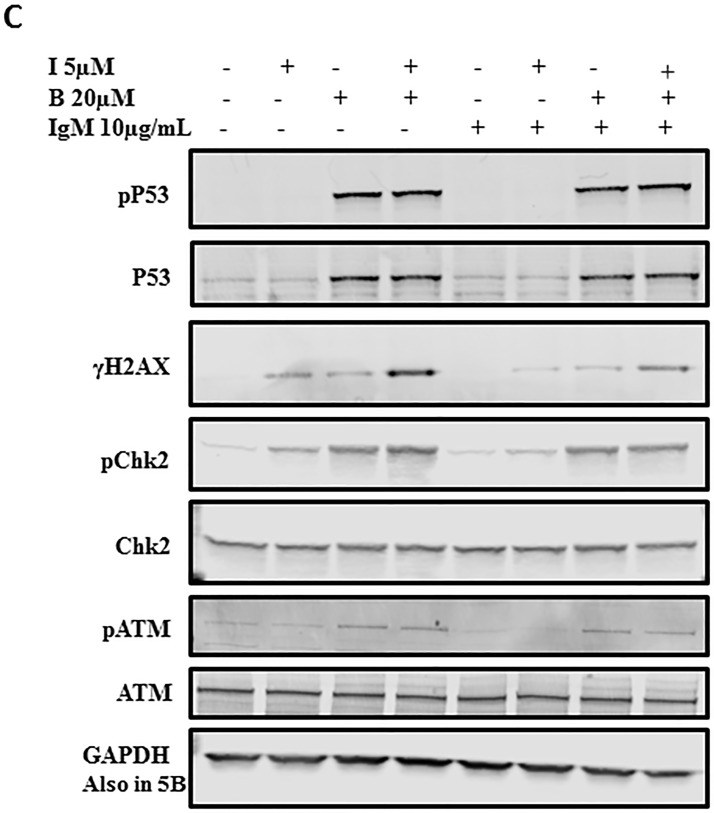
Idelalisib and bendamustine combination results in enhanced DNA damage response **A**.. Induction of γH2AX (measure of DNA damage response) after treatment with idelalisib, bendamustine, or the combination. Primary cells were treated with DMSO (D), idelalisib (I, 5 μM) alone, bendamustine (B, 20 μM) alone, or both idelalisib and bendamustine (5 μM + 20 μM, respectively) for 24 hours. The cells were then harvested, fixed with ethanol, incubated with goat serum for 1 hour, and probed with the primary antibody γH2AX. Cells were washed and then co-incubated with PI and DNase-free RNase and analyzed for fluorescence signal on flow cytometry for DNA damage response. Fluorescent cells, with an upward shift were marked as γH2AX-positive cells. **B**. Data similar to that in Figure 2A were collected from 5 patients with CLL (CLL514, CLL455, CLL327, CLL137, and CLL103). Horizontal bar represents median value. The significance of the differences between treatment groups was determined by a paired 2-tailed Student *t*-test. **C**.. Effect of idelalisib, bendamustine, and combination treatment on DNA damage proteins in CLL cells. Primary cells were either unstimulated or stimulated with αIgM (10 μg/mL) for 30 minutes. The cells were then treated with DMSO, idelalisib alone (5 μM), bendamustine (20 μM) alone, or both idelalisib and bendamustine (5 μM + 20 μM, respectively) for 24 hours. Cells were harvested, lysed and analyzed using immunoblot technique to detect the protein levels of phospho- and total-p53, γH2AX, phospho- and total-Chk2, and phospho- and total-ATM. Glyceraldehyde 3-phosphate dehydrogenase (GAPDH) was used as a control for equal protein loading.

Bendamustine is an alkylating agent known to induce DNA damage response. γH2AX is a prominent marker of DNA damage response [17] and could be measured using flow-cytometry assay as shown in the plots (Figure 2A). CLL primary cells were either untreated or treated with idelalisib alone, bendamustine alone or combination. Control i.e. DMSO only treated CLL lymphocytes showed a minimal positivity for γH2AX. Compared to the control, single agent idelalisib and single agent bendamustine demonstrated increased γH2AX. Furthermore, when both drugs were incubated simultaneously, the combination treatment significantly enhanced DNA damage response, according to both flow cytometry (Figure 2B) and immunoblot analysis (Figure 2C). When CLL primary cells were stimulated with αIgM, there was a slight decrease in DNA damage response (Figure 2C).

We further evaluated the effects of these agents on other proteins involved in DNA damage/repair signaling pathways. Compared to the control, bendamustine alone increased phosphorylation of ATM(Ser1981) and Chk2(Thr68), an effect that was further enhanced by combination treatment. Additionally, bendamustine alone and in combination with idelalisib resulted in stabilization of p53 protein, marked by phosphorylation of p53(Ser15). Overall, these observations indicate that idelalisib enhanced the DNA-damage response elicited by bendamustine.

### Idelalisib treatment decreases global RNA synthesis and impacts short-lived mRNAs

**Figure 3:**
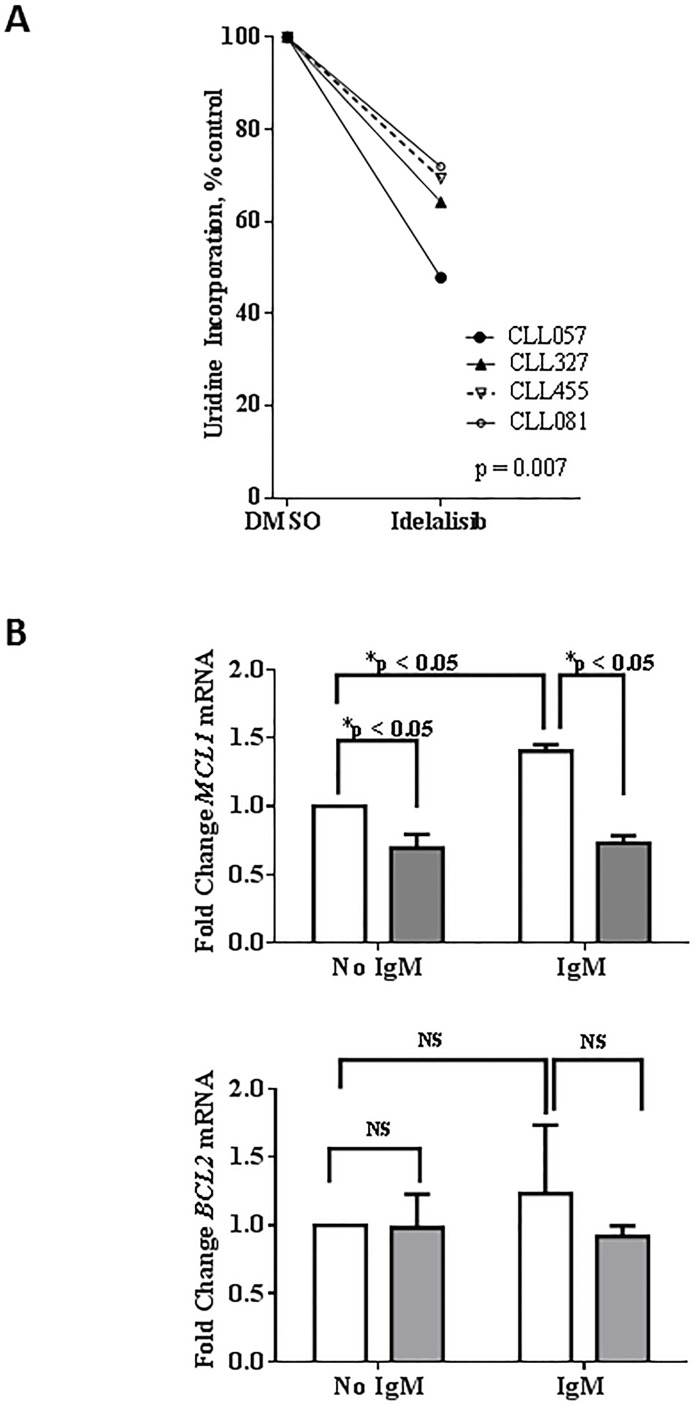
Effect of idelalisib on global RNA synthesis and ***MCL1*** transcript levels. **A**. Inhibition of global RNA synthesis by idelalisib in primary CLL cells. CLL peripheral blood mononuclear cells isolated from patient samples were treated with dimethyl sulfoxide (DMSO) or with idelalisib (5 μM) for 24 hours. The cells were co-incubated with [5,6-^3^H]-uridine for 30 minutes before harvesting, and the amount of radioactivity incorporated into the cells was measured using a scintillation counter. Radioactivity was calculated and normalized to the DMSO control. Experiments using 4 patient samples (CLL057, CLL327, CLL455, and CLL081) were performed in triplicate. The significance of the differences between treatment groups was determined by a paired 2-tailed Student *t*-test. **B**. Decreased *MCL1* mRNA expression, with no change in *BCL2* mRNA expression, in idelalisib-treated CLL cells. Primary cells were either unstimulated or stimulated with αIgM (10 μg/mL) for 30 minutes. Cells then were treated with DMSO or idelalisib (5 μM) for 24 hours and total RNA was extracted and quantified. Isolated RNA was analyzed by real-time RT-PCR with primers and probes for *MCL1* and *BCL2* mRNA transcripts. *MCL1* and *BCL2* gene expression levels were measured and normalized to the *18S* ribosomal RNA as an endogenous control, and each experiment was normalized to the DMSO control. The results are shown for 8 patient samples (CLL075, CLL483, CLL454, CLL293, CLL068, CLL354, CLL653, and CLL516). Each bar represents mean +/- SEM for the 8 patient samples. The p values were calculated using paired *t*-test. Abbreviations: NS, not significant.

Consistent with previous studies of PI3Kα and β isoforms, [18] we found that idelalisib-treated cells had significantly less global RNA synthetic capacity than control cells (47%-71% decrease; *n* = 4; Figure 3A). We next examined which particular mRNA transcripts are being depleted with idelalisib treatment. In CLL, *MCL1* and *BCL2* are the members of the BCL2 family antiapoptotic proteins, [19]. Our results showed that, in unstimulated cells, idelalisib treatment significantly decreased *MCL1* mRNA expression (by 30%); Of note, *MCL1* mRNA expression was increased by IgM stimulation, but decreased again with idelalisib treatment (Figure 3B; upper panel). In contrast, *BCL2* mRNA expression did not change significantly (Figure 3B; lower panel).

### Idelalisib treatment decreases Mcl-1 but not Bcl-2 protein levels in CLL cells

**Figure 4:**
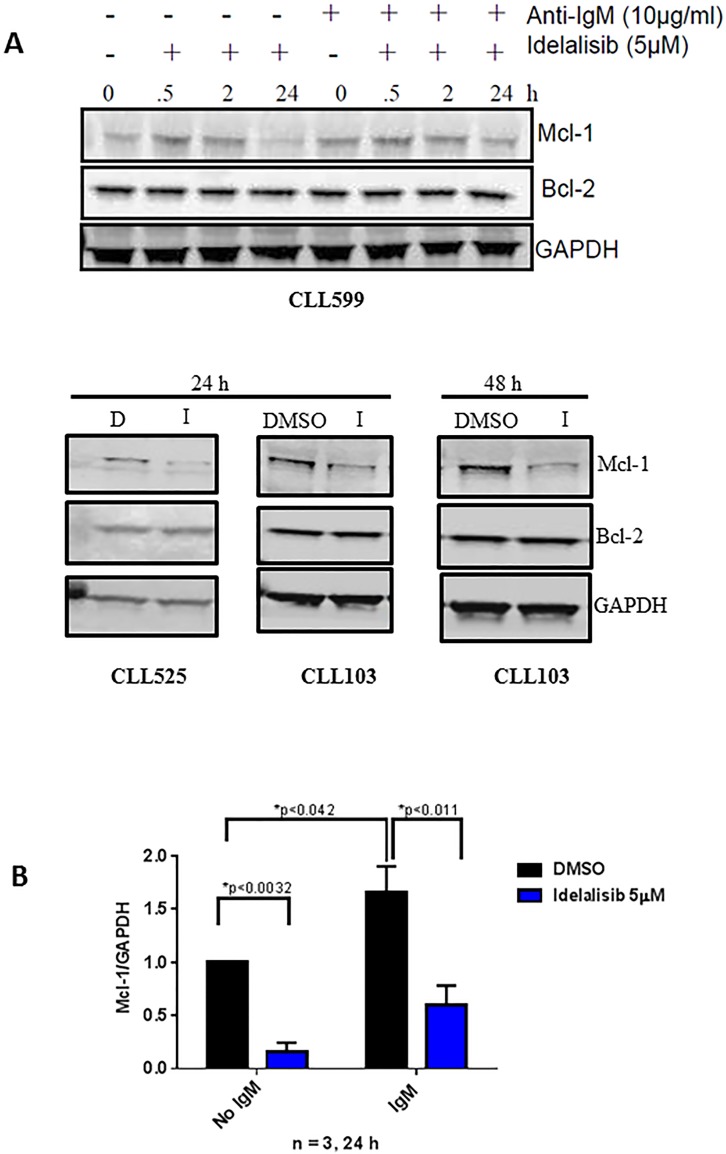
Effect of idelalisib on Mcl-1 protein levels **A**. Decreased Mcl-1 protein expression, with no change in Bcl-2 protein expression, in idelalisib-treated CLL cells. Primary cells either unstimulated or stimulated with αIgM (10 μg/mL) for 30 minutes and then were treated with DMSO (D) or idelalisib (I, 5 μM) for different times (0, 0.5, 2, 24 hours [h]). Cells were harvested, and protein lysates were analyzed using immunoblots to detect the protein levels of MCL1 and BCL2. GAPDH was used as a control to check for equal protein loading. **B**. Immunoblots for three patient samples (from 4A) were quantified by measuring the ratios of Mcl-1 to GAPDH for idelalisib-treated samples, and these values were normalized to the DMSO control. The p values were calculated using paired *t*-test.

In order to examine if the transcription results obtained above are in conjunction with translation, we evaluated the protein levels of Mcl-1 and Bcl-2. CLL primary cells from one patient sample (CLL599) was either unstimulated or stimulated with αIgM and treated with 5 μM of idelalisib for increasing time points (30 min, 2 h and 24 h) and the immunoblot technique was carried out to evaluate Mcl-1 and Bcl-2 proteins. Additional samples (CLL525 (24 h), CLL103 (24 h) and CLL103 (48 h)) were evaluated for the same proteins. Consistent with our mRNA data, idelalisib treatment decreased Mcl-1 protein but not Bcl-2 protein levels (Figure 4A).

### Effect of single agent bendamustine and in combination with idelalisib on Mcl-1 expression

**Figure 5:**
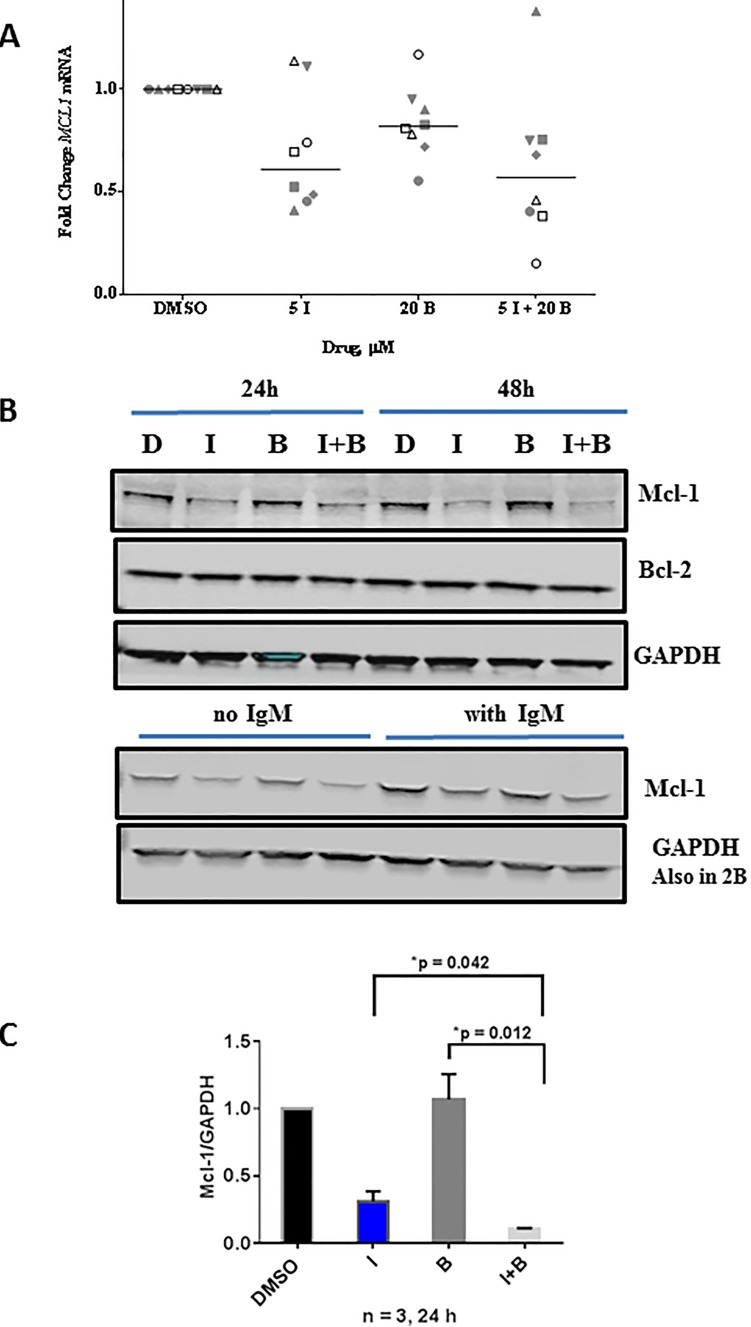
Comparison of single agent idelalisib, single agent bendamustine to combination regimen on ***MCL1*** transcript and Mcl-1 protein levels. **A**. Primary cells were treated with DMSO, idelalisib (I, 5 μM) or bendamustine (B, 20 μM) alone, or in combination. Cells were harvested, and total RNA was extracted and analyzed by real-time RT-PCR with primers and probe for the *MCL1* mRNA transcript. *MCL1* gene expression was measured and normalized to the *18S* ribosomal RNA as an endogenous control, and each experiment was normalized to the DMSO control. The data are from 8 CLL patients (CLL516, CLL068, CLL454, CLL483, CLL354, CLL203, CLL653, and CLL075). Horizontal bar represents median value. **B**. Effect of idelalisib and bendamustine combination treatment on apoptotic protein levels in primary CLL cells. Primary cells were treated with DMSO, idelalisib (I, 5 μM) or bendamustine (B, 20 μM) alone, or in combination (5 μM I + 20 μM B) for 24 hours and 48 hours. Cells were harvested, and protein lysates were analyzed using immunoblots to detect the protein levels of Mcl-1 and Bcl-2. GAPDH was used as a control for equal protein loading. The lower immunoblot represents cells treated with or without IgM (same extract used in Figure 2B) and tested for γH2AX. This immunoblot was from the same gel as in Figure 2B and hence same GAPDH is used. **C**. Immunoblots for three patient samples (from Figure 5B) were quantified by measuring the ratios of Mcl-1 and Bcl-2 to GAPDH for idelalisib- and bendamustine-treated samples, and these values were normalized to the DMSO control. Paired *t*-test was used to determine *p* values.

Single agent bendamustine also depleted *MCL1* mRNA levels in primary CLL cells (*n* = 8) however not to the extent of idelalisib induced depletion tested in the same samples (Figure 5A). When the protein levels were evaluated with bendamustine and bendamustine and idelalisib combination, in both unstimulated and αIgM stimulated cells (24 h and 48 h) there was no marked changes in Mcl-1 protein levels (Figure 5B). On same note, there were no significant changes in BCL2 mRNA (Supplemental Figure 1) or protein levels (Figure 5B). Quantitation of immunoblots for Mcl-1 protein revealed that although single agent bendamustine does not alter the Mcl-1 protein levels, this agent in combination with idelalisib does significantly enhance the depletion of this short-lived protein (Figure 5C).

### Impact of Mcl-1 loss on bendamustine and combination-induced cytotoxicity in MEFs

**Figure 6:**
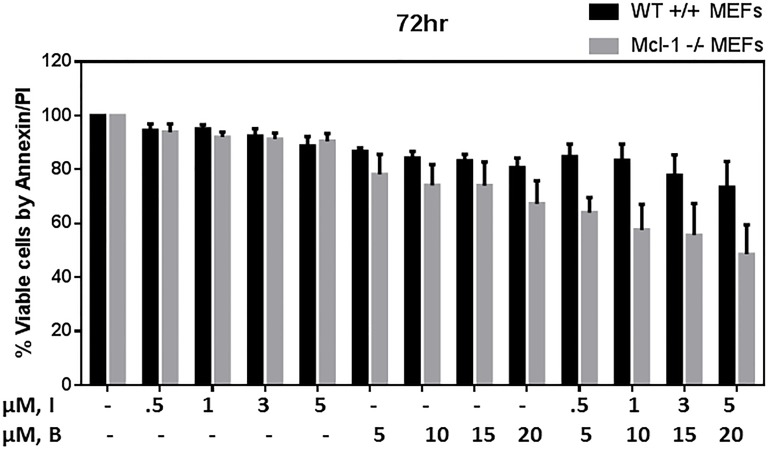
Modulation of Bendamustine-induced cytotoxicity in MEFs lacking ***MCL1***. Increased cytotoxicity in bendamustine-treated mouse embryonic fibroblasts (MEFs) lacking *MCL1* compared with wild-type (WT) MEFs. MCL1^wt/wt^ and MCL1^Δ/null^ MEFs were treated with DMSO, idelalisib (0.5 μM, 1 μM, 3 μM, 5 μM) or bendamustine (5 μM, 10 μM, 15 μM, 20 μM) alone, or a combination of idelalisib and bendamustine (0.5 μM +5 μM, 1 μM +10 μM, 3 μM +15 μM, or 5 μM +20 μM, respectively) for 72 hours. Cells were harvested, stained with Annexin V and propidium iodide (PI), and the amount of apoptotic cells in each treated sample was detected using flow cytometry. Experiments were done in triplicate, and the results show the averages ± SEM.

Mcl-1, an anti-apoptotic protein of Bcl-2 family is considered important for CLL cell survival. In addition, BCR activation enhances Mcl-1 protein levels and inhibition of BCR signaling with kinase inhibitors deplete Mcl-1. Consistently, our current results demonstrate that idelalisib inhibits Mcl-1 transcript and protein levels, with no differential effect with bendamustine. To directly assess the role of Mcl-1 in bendamustine-induced cytotoxicity, we tested the apoptotic response of isogenic MEFs (Supplemental Figures 2 and 3) treated with idelalisib, bendamustine, or combination. Bendamustine- (*p* = 0.021) or combination-treated (*p* = 0.017) MEFs lacking *MCL1* had significantly higher apoptotic cells compared to wild-type MEFs (Figure 6); indicating that the combination regimen potently downregulates Mcl-1 and thus enhance the apoptosis in CLL cells. This differential effect was not observed with idelalisib (*p* = 0.478).

## DISCUSSION

Bendamustine alone showed a dose- and time- dependent cytotoxicity of quiescent CLL lymphocytes (Figure 1B). The primary mechanism of cell death is damage of DNA, genotoxic stress, and apoptosis [14, 20]. While idelalisib-induced apoptosis was minor (Figure 1A), the couplet of bendamustine and idelalisib resulted in synergistic combination at many different concentrations (Figure 1D). This is in concert with a prior report where combination of idelalisib at 0.5 μM resulted in sensitization of CLL cells to bendamustine (25 μM) induced cell death [15]. Mechanistically, we provide two actions of idelalisib to enhance bendamustine's cytotoxic effect in CLL lymphocytes; first is an impact on DNA damage and repair response and second is a depletion of Mcl-1 protein. Down-regulation of CD69, a biomarker in CLL, by idelalisib has been previously shown to be responsible for sensitization of CLL cells to bendamustine [15].

Bendamustine is an established alkylating agent resulting in double strand breaks. It is 4-{5- [bis(2-chloroethyl)amino]-1-methyl-2-bezimidazolyl} butyric acid hydrochloride. The nitrogen mustard group of bendamustine is similar to that in chlorambucil [12, 21]. However, randomized study comparing chlorambucil to bendamustine suggested increased overall response rate with bendamustine [22]. Nitrogen mustard induced damage results in monoadducts, biadducts, and intra- and interstrand cross-links in DNA. As a consequence of this damage response, as expected, bendamustine resulted in induction of gammaH2AX (Figure 2A) along with other hallmark features such as Chk2 phosphorylation (Figure 2B). Paradoxically, and not expected, treatment of CLL cells to idelalisib alone also resulted in initiation of DNA damage response (Figure 2A and 2B and unpublished data). Additionally, in combination with bendamustine, idelalisib resulted in stabilization of p53 protein, marked by phosphorylation of p53(Ser15), Chk2 phosphorylation, and induction of gammaH2AX; all markers of DNA damage. Phosphorylation of p53, Chk2, and H2AX are mediated through active ATM. [14, 23, 24] It is worthy to mention that none of the patient samples studied had 11q or 17p deletion, the sites for ATM and p53, respectively.

The Mcl-1 molecule [25] is an important and bonafide prosurvival protein for CLL. [26] However, as we found in our study, Mcl-1 has been shown to be involved in DNA damage and repair. [27] Other investigators have reported that cytotoxic DNA-damaging agents that cause an early apoptosis response lead to enhanced *MCL1* gene expression in a p53-independent manner. [27–30] Particularly, Mcl-1 is linked to regulating cell-cycle progression and is partially mediated through PCNA, interactions with CDK1, and ATR-dependent activation of Chk1 following DNA damage. [31–33] Overall, Mcl-1 is highly overexpressed in many human cancers, is manipulated by malignant cells to escape apoptosis regulation, and has a unique role in the DNA damage response.

Mcl-1 is a cytosolic protein and in this location it has been established to inhibit endogenous or drug-induced apoptosis. During DNA damage response, it is translocated to nucleus [27, 33–35]. Nuclear Mcl-1 interacts with many DNA repair/damage proteins such as H2AX, Nbs1, Ku70, and co-localizes with 53BP1 and is involved in homologous recombination pathway [35].

In addition to Mcl-1, several other pro and anti-survival members of the Bcl-2 family have been shown to participate in DNA repair. Pro-apoptotic protein Bid binds to RPA protein [36]. Bcl-2 has been shown to impact DNA double strand break repair [37] by inhibition of recruitment of Mre11 complex to the site of double strand breaks in the DNA [38]. Collectively, these reports establish mechanistic role of Bcl-2 family members in DNA damage repair which is in addition to the pro and anti-survival manifestations of these proteins.

Both *MCL1* transcript and Mcl-1 protein are short-lived due to the presence of ARE-rich regions and PEST domains in transcript and proteins, respectively. Hence, even a short-term block or inhibition in transcription or protein translation results in a decline in *MCL1* transcript and Mcl-1 protein levels. Furthermore, the stability of this protein is impacted by several pathways. Erk, a kinase involved in cancer cell survival, phosphorylates Mcl-1 which prevents proteasomal degradation of Mcl-1 [39, 40]. Proteasomal degradation of Mcl-1 is dependent on phosphorylation of this protein by GSK3β [41]. However, phosphorylation of GSK3β by Akt mitigates activity of GSK3β resulting in prolonged presence of Mcl-1 [42]. Both Akt and Erk are induced through BCR pathway [43, 44]. Corollary to this phenomenon, inhibition of BCR axis by PI3K/Akt inhibitors modulates Akt and Erk activities [2, 45] which may impact Mcl-1 protein stability and may induce degradation of this protein.

Down regulation of MCL1 transcript and protein levels was observed after treatment of CLL cells during *in vitro* incubations (Figures 3 and 4) of CLL cells with idelalisib. In concert to this observation, clinically, treatment of patients with idelalisib showed lowered expression of Mcl-1 protein in circulating CLL lymphocytes after 2, 4, and 12 weeks of idelalisib intake (Yang, Modi unpublished). Other small molecule targeted inhibitors of PI3K [45], BCR pathway [46], and BTK [47] also result in depletion or decrease in intracellular levels of Mcl-1 protein in CLL lymphocytes. Overall, decrease in Mcl-1 protein expression is a pharmacodynamic phenomenon in CLL cells after treatment with BCR pathway inhibitors.

The phase II clinical trial of bendamustine and rituximab in relapsed/refractory CLL disease, demonstrated 60% overall response-rate [48]. Although the overall response rate and CR rate are lower than the FCR therapy, this regimen is preferred for low untoward toxicity profile. To increase response rates, fludarabine [49], ibrutinib (HELIOS trial; [50]), and idelalisib [51] have been added to the bendamustine and rituximab couplet regimen. The HELIOS trial was a randomized study where bendamustine and rituximab combination was compared with the triplet of bendamustine and rituximab with ibrutinib. Addition of ibrutinib led to significant benefit for overall response rate and progression-free survival. Importantly, the triple combination did not add any cumulative toxicities [50]. Similar to this combination, preliminary and interim report also suggested that idelalisib improves progression-free survival when added to the bendamustine and rituximab couplet [51, 52]. These trial results clearly suggest clinical benefit of adding BCR pathway inhibitor to bendamustine plus rituximab chemoimmunotherapy.

In conclusion, we demonstrate that the addition of idelalisib synergistically benefits bendamustine-induced cytotoxicity in CLL lymphocytes. Furthermore, idelalisib-mediated DNA damage response and decline in *MCL1* mRNA and Mcl-1 protein levels may in-part be mechanisms of this synergistic cooperation.

## MATERIAL, PATIENTS, AND METHODS

### Patient sample collection

Peripheral blood was obtained from CLL patients (Supplemental Table 1) who had given written informed consent in accordance with the Declaration of Helsinki. The study protocol was approved by the Institutional Review Board of The UT MD Anderson Cancer Center. Peripheral blood mononuclear cells were isolated by Ficoll-Hypaque density gradient centrifugation (Atlanta Biologicals, Norcross, GA). Cells (1×10^7^/mL) were cultured in RPMI-1640 medium with 10% autologous patient serum and were freshly used. For *in vitro* BCR activation, CLL cells were stimulated with polyclonal goat F(ab’)2 fragments of human IgM (MP Biomedicals, Santa Ana, CA).

### Mouse embryo fibroblast cell line

Mouse embryonic fibroblasts (MEFs), wild-type and *MCL1* deficient were generously provided by Dr. Joseph T. Opferman at St. Jude Children's Research Hospital (Memphis, TN) [25] Both cell lines are Simian virus (SV40)-transformed and the cells were maintained in Dulbecco modified Eagle medium with L-glutamine (DMEM; Invitrogen) media supplemented with 10% fetal bovine serum (FBS; Invitrogen), Pen/Strep, L-Glut, and non-essential amino acids (NEAA; GIBCO). Presence or absence of Mcl-1 protein in these cells were confirmed by Dr. Opferman's group as well as by our group using immunoblots. Cell lines were periodically tested for *Mycoplasma* contamination using a MycoTect kit (Invitrogen). All experiments were conducted in cell passages less than 15 and were maintained at a logarithmic growth concentration between 10^5^ cells/mL and 10^6^ cells/mL with 80% confluency as determined by a Coulter channelyzer with less than 10% endogenous cell death confirmed by flow cytometry.

### Drugs

Idelalisib (GS-1101 or CAL-101) was provided by Gilead Sciences, Inc., (Foster City, CA). Bendamustine hydrochloride was originally obtained from Cephalon (Frazer, PA; now Teva Pharmaceuticals Industries, Ltd., Petah Tikva, Israel) and was later purchased from Selleckchem (Houston, TX). Both drugs were used in micromolar concentrations that were chosen on the basis of reported plasma concentrations of the free drug in patients. [1, 2, 5, 12, 14, 20] For bendamustine we used generally 20 μM exogenous drug. Bendamustine peak levels are 28 μM [53] or 20 - 24 μM at 90 and 120 mg/m^2^ [54, 55]. For idelalisib, we generally used 20 μM. Because more than 84% of idelalisib binds to human plasma proteins [1], only 16% of the free drug is available to the cells during *in vitro* culture conditions. Hence, exogenous addition of 5 μM idelalisib to *in vitro* culture may result in 0.8 μM free idelalisib available for activity. Such free-drug levels in plasma are achieved during idelalisib therapy.

### Cytotoxicity assays

For apoptotic assay, primary CLL cells were untreated or treated with idelalisib, bendamustine, or combination and stained with Annexin V and propidium iodide and counted using flow cytometry, as described previously. [56] Cells in all three quadrants (early apoptosis, late apoptosis, and necrosis) were included to obtain percent total cell death.

### γH2AX staining

The cells obtained before and after incubation with drugs were washed with PBS and fixed in 6 mL ice-cold 70% ethanol and analyzed for H2AX phosphorylation on the flow cytometry. The cells were then fixed with 4% fresh paraformaldehyde/PBS (pH 7.4) at room temperature for 10 min. After couple of washes with BSA/PBS, the cells were blocked with 5% goat serum for an hr. This was followed by incubation with anti-phospho-Histone H2AX (Ser139) mouse monoclonal antibody, clone JBW301, FITC conjugate (16-202A; Upstate, Billerica, MA) for 2 hr. The labeled cells were washed and resuspended in PBS containing the counterstain propidium iodide (15 μg/mL) and RNAase (Roche, South San Francisco, CA) (2.5 μg/mL) and incubated in dark for 5 min before analysis using FACScalibur (BD Biosciences, San Jose, CA). Data were expressed as fold increase of H2AX phosphorylation. In addition to flow cytometry assay, immunoblot assays were also performed to test for H2AX phosphorylation.

### Immunoblot analyses

Extracts from cell lysates were quantitated for protein concentration using a DC protein assay kit (Bio-Rad Laboratories, Hercules, CA, USA), loaded and transferred to nitrocellulose membranes (GE Osmonics Labstore, Minnetonka, MN, USA). Membranes were blocked for 1 h in licor blocking buffer, incubated with primary antibodies overnight at 4°C against the following: After washing with PBS-Tween-20, membranes were incubated with infrared-labeled secondary antibodies (LI-COR Inc., Lincoln, NE, USA) for an hour, scanned and visualized using LI-COR Odyssey Infrared Imager. [14] Antibodies for specific proteins and their catalog numbers were used and are listed in the table (Supplemental Table 2).

### RNA synthesis assay

Primary CLL cells were either untreated or treated with idelalisib for the indicated time. For the last 30 min, the cells were incubated with [5,6-^3^H]-uridine (1.0 mCi/mL stock; Moravek Biochemicals, Brea, CA), and the radioactive counts were measured by scintillation counter. [14] Each treatment was done in triplicate and data were presented as percent of control where control is time-matched untreated CLL cells.

### Transcript level measurements

CLL lymphocytes were treated with DMSO or with idelalisib alone, bendamustine alone, or two drugs together for 24 hours. TaqMan real-time reverse transcription polymerase chain reaction assay was used to measure *BCL2* and *MCL1* transcript levels, which were normalized to *18S* ribosomal RNA as an endogenous control. [56]

### Statistical analysis

Paired 2-tailed Student *t*-tests were performed using Prism-6 software (GraphPad Software, Inc., La Jolla, CA). For combination treatment, fractional analysis was used to determine whether the combination led to less than, equal to, or more than the additive effect on inducing apoptosis. [57] CalcuSyn software (CompuSyn Inc., Paramus, NJ) was used to determine the combination index.

### Authorship contribution

P.M. designed the experiments, performed the experiments, analyzed the results, and wrote the manuscript. K.B. directed P.M. in the laboratory and reviewed the manuscript. Q.Y. assisted in experimental planning and reviewed the manuscript. M.J.K. and W.G.W. identified patients to obtain peripheral blood samples, provided clinical and patient-related input, and reviewed the manuscript. V.G. conceptualized and supervised the research, obtained funding, analyzed the data, and wrote and reviewed the manuscript.

## SUPPLEMENTARY MATERIALS FIGURES AND TABLES


