# Supplementary material for: Idelalisib and bendamustine combination is synergistic and increases DNA damage response in chronic lymphocytic leukemia cells
Source: Oncotarget. 2017 Feb 7;8(10):16259–74. doi: 10.18632/oncotarget.15180 (PMC5369961; doi:10.18632/oncotarget.15180)
Supplement: Supplementary file 1 [file oncotarget-08-16259-s001.pdf]

Idelalisib and bendamustine combination is synergistic and increases DNA damage response in chronic lymphocytic leukemia cells

Supplementary Material

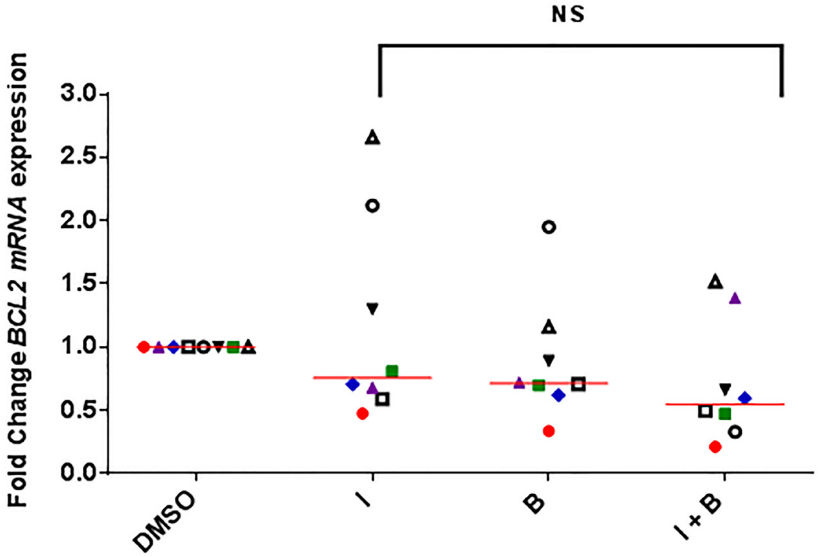

Supplemental Figure 1. Effect of idelalisib and bendamustine combination treatment on *BCL2* mRNA expression in primary CLL cells.

Primary cells were treated with dimethyl sulfoxide (DMSO), idelalisib (I, 5 µM) alone, bendamustine (B, 20 µM) alone, or a combination of idelalisib and bendamustine (5 µM + 20 µM, respectively) for 24 hours. Cells were harvested, and total RNA was extracted and quantified. Isolated RNA was analyzed by real-time reverse transcription polymerase chain reaction with primers and probes for *BCL2* mRNA transcript. *BCL2* mRNA levels were measured and normalized to the *18S* ribosomal RNA as an endogenous control, and each experiment was normalized to the DMSO control. The figure represents 8 CLL patients (CLL516, CLL068, CLL454, CLL483, CLL354, CLL203, CLL653, and CLL075). A paired 2-tailed Student t-test was performed for these patient samples, and the mRNA levels were not significantly different (NS). Horizontal bar represents median value.

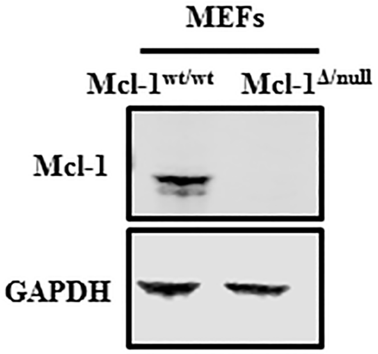

Supplemental Figure 2. MCL-1 protein expression in MCL-1$^{wt/wt}$ and MCL-1$^{\Delta/null}$ mouse embryonic fibroblasts (MEFs).

MCL1$^{wt/wt}$ and MCL1$^{\Delta/null}$ MEFs were cultured in DMEM medium with 10% serum and were harvested and protein lysates were analyzed using immunoblots to detect the MCL1 protein levels to verify loss of MCL-1 protein in deficient MEFs. Glyceraldehyde 3-phosphate dehydrogenase (GAPDH) was used as a control for equal protein loading. A mouse-specific antibody detected the expression of mouse MCL1 in wild-type and MCL1 deleted isogenic cell lines. Abbreviation: WT, wild-type.

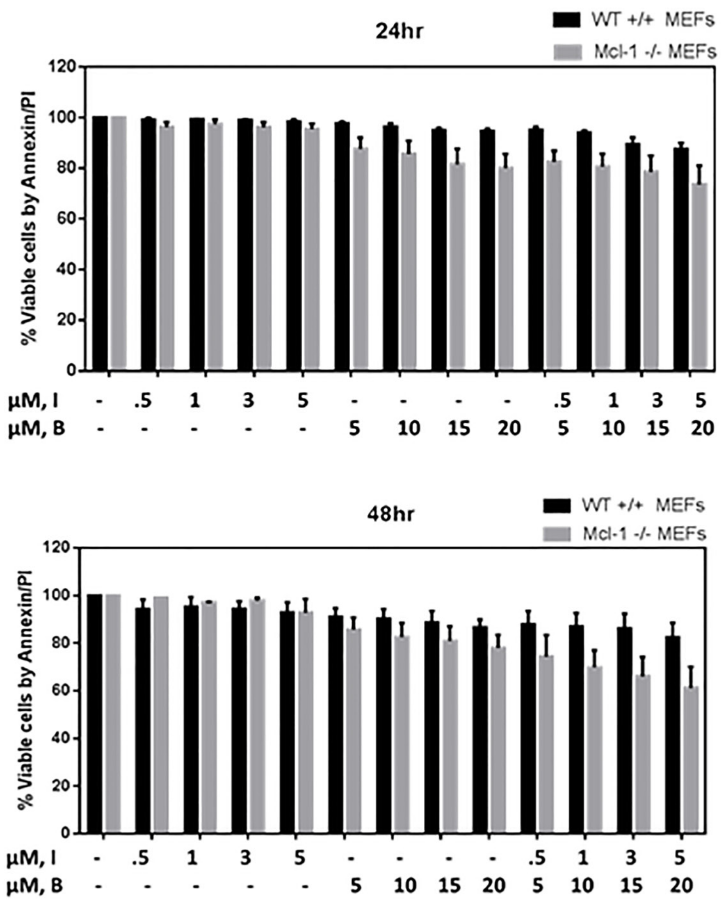

Supplemental Figure 3. Dose- and time-dependent decreases in cell viability in MCL1$^{wt/wt}$ and MCL1$^{\Delta/null}$ mouse embryonic fibroblast (MEFs) lines treated with a combination of idelalisib (I) and bendamustine (B).

MCL1$^{wt/wt}$ and MCL1$^{\Delta/null}$ MEFs were treated with dimethyl sulfoxide (DMSO), idelalisib (0.5 µM, 1 µM, 3 µM, or 5 µM) alone, bendamustine (5 µM, 10 µM, 15 µM, or 20 µM) alone, or a combination of idelalisib and bendamustine (0.5 µM + 5 µM, 1 µM + 10 µM, 3 µM + 15 µM, or 5 µM + 20 µM, respectively) for 24 and 48 hours (h). Cells were harvested and then stained with Annexin V followed by propidium iodide (PI). The levels of apoptosis for each treatment were detected using flow cytometry. Experiments were done in triplicate, and the results show the averages $\pm$ SEM.
